# Supplementary material for: A high-resolution mRNA expression time course of embryonic development in zebrafish
Source: eLife. 2017 Nov 16;6:e30860. doi: 10.7554/eLife.30860 (PMC5690287; doi:10.7554/eLife.30860)
Supplement: Supplementary file 6. [file elife-30860-supp6.zip › biolayout-clusters-files/Cluster120-genes.html]

Cluster120


# Cluster120: Genes

| Ensembl ID | Gene Name | Chr | Start | End | Biotype |
| --- | --- | --- | --- | --- | --- |
| ENSDARG00000070485 | apitd1 | 6 | 45932800 | 45935118 | protein\_coding |
| ENSDARG00000012113 | c19h1orf109 | 19 | 48140594 | 48144828 | protein\_coding |
| ENSDARG00000013351 | cirbpb | 22 | 18517032 | 18521296 | protein\_coding |
| ENSDARG00000056914 | emg1 | 16 | 17346512 | 17350209 | protein\_coding |
| ENSDARG00000003527 | mak16 | 8 | 45387035 | 45408308 | protein\_coding |
| ENSDARG00000045373 | pabpn1 | 24 | 14414909 | 14433140 | protein\_coding |
| ENSDARG00000035264 | riok2 | 5 | 41039540 | 41044905 | protein\_coding |
| ENSDARG00000042864 | rpl7l1 | 20 | 36772204 | 36776354 | protein\_coding |
| ENSDARG00000097102 | si:ch73-281n10.2 | 15 | 5910559 | 5913663 | protein\_coding |
| ENSDARG00000015759 | tspan7 | 14 | 41102224 | 41111138 | protein\_coding |
| ENSDARG00000078606 | zgc:194224 | 20 | 3874445 | 3882256 | protein\_coding |
